# Gene discovery in EST sequences from the wheat leaf rust fungus *Puccinia triticina *sexual spores, asexual spores and haustoria, compared to other rust and corn smut fungi

**DOI:** 10.1186/1471-2164-12-161

**Published:** 2011-03-24

**Authors:** Junhuan Xu, Rob Linning, John Fellers, Matthew Dickinson, Wenhan Zhu, Ivan Antonov, David L Joly, Michael E Donaldson, Tamar Eilam, Yehoshua Anikster, Travis Banks, Sarah Munro, Michael Mayo, Brian Wynhoven, Johar Ali, Richard Moore, Brent McCallum, Mark Borodovsky, Barry Saville, Guus Bakkeren

**Affiliations:** 1 Pacific Agri-Food Research Centre, Agriculture & Agri-Food Canada, Summerland, BC, V0H 1Z0, Canada; 2 USDA-ARS-HWWGRU, Manhattan, KS 66506, USA; 3 School of Biosciences, University of Nottingham, Loughborough, UK; 4 Dept. Biomedical Engineering and Computational Science and Engineering Division, Georgia Institute of Technology, Atlanta, GA 30332-0535, USA; 5 Environmental & Life Sciences Graduate Program, Trent University, Peterborough, ON, K9J 7B8, Canada; 6 Institute for Cereal Crops Improvement, George S. Wise Faculty of Life Sciences, Tel Aviv University, Tel Aviv 69978, Israel; 7 Cereal Research Centre, Agriculture & Agri-Food Canada, Winnipeg, MB, R3T 2M9, Canada; 8 Michael Smith Genome Sciences Centre, Vancouver, BC, V5Z 4E6, Canada; 9 Forensic Science Program Trent University, Peterborough, ON, K9J 7B8, Canada; 10 Bee Biology & Systematics Laboratory, USDA-ARS, N. Logan, UT 84341, USA

## Abstract

**Background:**

Rust fungi are biotrophic basidiomycete plant pathogens that cause major diseases on plants and trees world-wide, affecting agriculture and forestry. Their biotrophic nature precludes many established molecular genetic manipulations and lines of research. The generation of genomic resources for these microbes is leading to novel insights into biology such as interactions with the hosts and guiding directions for breakthrough research in plant pathology.

**Results:**

To support gene discovery and gene model verification in the genome of the wheat leaf rust fungus, *Puccinia triticina *(*Pt*), we have generated Expressed Sequence Tags (ESTs) by sampling several life cycle stages. We focused on several spore stages and isolated haustorial structures from infected wheat, generating 17,684 ESTs. We produced sequences from both the sexual (pycniospores, aeciospores and teliospores) and asexual (germinated urediniospores) stages of the life cycle. From pycniospores and aeciospores, produced by infecting the alternate host, meadow rue (*Thalictrum speciosissimum*), 4,869 and 1,292 reads were generated, respectively. We generated 3,703 ESTs from teliospores produced on the senescent primary wheat host. Finally, we generated 6,817 reads from haustoria isolated from infected wheat as well as 1,003 sequences from germinated urediniospores. Along with 25,558 previously generated ESTs, we compiled a database of 13,328 non-redundant sequences (4,506 singlets and 8,822 contigs). Fungal genes were predicted using the EST version of the self-training GeneMarkS algorithm. To refine the EST database, we compared EST sequences by BLASTN to a set of 454 pyrosequencing-generated contigs and Sanger BAC-end sequences derived both from the *Pt *genome, and to ESTs and genome reads from wheat. A collection of 6,308 fungal genes was identified and compared to sequences of the cereal rusts, *Puccinia graminis *f. sp. *tritici *(*Pgt*) and stripe rust, *P. striiformis *f. sp. *tritici *(*Pst*), and poplar leaf rust *Melampsora *species, and the corn smut fungus, *Ustilago maydis *(*Um*). While extensive homologies were found, many genes appeared novel and species-specific; over 40% of genes did not match any known sequence in existing databases. Focusing on spore stages, direct comparison to *Um *identified potential functional homologs, possibly allowing heterologous functional analysis in that model fungus. Many potentially secreted protein genes were identified by similarity searches against genes and proteins of *Pgt *and *Melampsora *spp., revealing apparent orthologs.

**Conclusions:**

The current set of *Pt *unigenes contributes to gene discovery in this major cereal pathogen and will be invaluable for gene model verification in the genome sequence.

## Background

*Puccinia triticina *(*Pt*) has a complex life cycle which includes five different spore types and two hosts: wheat (*Triticum aestivum *L.) and meadow rue (*Thalictrum speciosissimum *L.). The latter plant is the so-called alternate host on which the fungus completes its sexual stage [1-3]. Sex is not essential and infection of and spread on wheat through re-infection constitutes the asexual cycle. The brown-coloured urediniospores from which the rust got its name, are the asexual infectious propagules. They are easily carried long distances by prevailing winds and can lead to epidemics. Early processes in infection include urediniospore attachment, germination and the formation of a germtube. The tips of germtubes differentiate into appressoria which develop over stomatal lips and entry into the substomatal cavity is gained forcibly by turgor pressure. Within 24 hrs after spore germination, a haustorial mother cell is formed adjacent to a plant cell within the cavity and cell wall penetration takes place. Subsequent invagination of the host plasmalemma results in the first intimate contact. Thereafter, a microscopically visible haustorial interface surrounding the mature feeding structure, is produced, likely made up of both fungal and host material [4]. This interface is critical in governing protein and metabolite traffic [5,6] since haustoria are thought to secrete a suite of proteins, some of which are aimed at suppressing host defence responses that may be triggered by the fungus when it penetrates the plant cell wall or at establishing the feeding interaction. In compatible interactions, the fungus colonizes the plant and within 7 days can produce uredinia (pustules) containing asexual urediniospores which are released and give rise to new rounds of infection.

On senescing wheat plants, uredinia respond to cues and switch to producing black teliospores. These are survival propagules with a complex, multi-layered wall and no vacuoles. They contain lipid droplets and glycogen-like material [7,8]. Teliospores are primarily 2-celled with each cell containing two haploid nuclei that have paired, if not fused to form the diploid state [8]. They often appear in low numbers on the lower parts of the plant, including the stem. Under the proper conditions, the teliospore germinates and a metabasidium forms, generally from both cells, in which meiosis occurs and on which four haploid basidiospores develop. A third mitotic division occurs resulting in basidiospores having two nuclei though being monokaryons, containing two nuclei of the same type [9]. These basidiospores are ephemeral and can be dispersed to infect the alternate host. This infection occurs via direct penetration of the plant epidermis and the subsequent production of monokaryotic (M-) haustoria. These structures are extensions of the intercellular hyphae which penetrate the plant cells in a manner reminiscent of that described for the corn smut fungus, *Ustilago maydis *[10]. Neither infection shows the morphological specialization seen for the *P. triticina *dikaryotic (D-) haustorium [11]. Upon establishing a feeding relationship and presumably suppressing alternate host defence responses, the monokaryotic hyphae yield specialized pycnia which generate pycniospores embedded in nectar. Because they originated from haploid meiotic products, the pycniospores represent different mating types and can cross-fertilize, often through the action of insects attracted by the nectar. After fertilization, that is, the fusion of one pycniospore to a receptive hypha in the pycnium of a different mating type followed by nuclear transfer, the newly formed dikaryon undergoes developmental reprogramming. The resultant mycelium traverses the leaf and forms aecia on the underside in which dikaryotic aeciospores develop [7]. Aeciospores are dispersal propagules which will infect the primary wheat host.

The generation of genomic resources for the cereal rust fungi is gearing up. A draft genome sequence for the related stem rust fungus, *P. graminis *f. sp. *tritici *(*Pgt*) was released in 2007, a rough draft for *Pt *was released in 2009, and genome sequences are being generated for the related wheat stripe rust fungus, *P. striiformis *f. sp. *tritici *(*Pst*; http://www.broadinstitute.org/annotation/genome/puccinia_group/MultiHome.html). The generation of Expressed Sequence Tags (ESTs) is essential for proper gene prediction in genomes. Previous *Pt *EST libraries contributed to gene discovery and stage-specific expression analyses [12-14]. Similar EST collections were generated from other *Puccinia *species [15-21]. However, all these studies were focused on urediniospores and the wheat infection cycle. There is no molecular data on genes involved in the sexual stages in the rusts. Unique to this study, we surveyed three other spore types representing the sexual stage: teliospores, pycniospores and aeciospores. We found that each spore type yielded rather specific EST sequences and that pycniospores and teliospores in particular seemed to express a unique set, when compared to all other sampled stages.

In addition, we generated and analysed ESTs from two sets of isolated haustoria and from the germinated urediniospore stage. Haustoria ESTs included a large set of unique sequences of unknown function. In total, over 17,000 new ESTs, of which many appeared spore-specific, were added to our existing collection of 25,558 sequences and compiled into a single unigene set. Subsequently, fungal genes were predicted using bioinformatic algorithms trained on *Pgt *sequences, and were then compared to *Pgt *and model basidiomycete plant pathogen, *U. maydis *genomic resources. Notably, comparison to *U. maydis *revealed interesting leads for research into spore biology.

## Results and Discussion

### Life cycle stage-specific cDNA libraries

*Three different sexual stage spore types *- Minute amounts of nectar, containing pycniospores of different mating types, as well as dikaryotic aeciospores, were collected from *Thalictrum *pycnia and aecia residing on the upper and abaxial side of leaves, respectively. Both life cycle stages were from the same isolate, but different from race 1. Race 1 teliospores were induced on senescing wheat and small amounts of dormant spores cleanly dissected from telial pustules. From these three stages, only nanogram-amounts of total RNA could be obtained. From the aeciospore material, cDNA library PT029 was constructed and the pycniospore RNA resulted in library PT030. The RNA from the teliospores produced cDNA library PT031.

*Isolated haustoria *-The haustorium is an important structure involved in bidirectional traffic of proteins and uptake of nutrients and ESTs might reveal expression of genes involved in host defence suppression and fungal feeding process. Moreover, haustoria are expected to produce effectors, some of which might have avirulence functions as has been shown to occur in other systems [22-24]. We therefore isolated haustoria from heavily-infected wheat leaves and produced two cDNA libraries from two different isolates: library designation PT033 (isolate PRTUS3) and PTDH (isolate WBRS-97-3; see Methods).

*Germtube *- Rust isolate WBRS-97-3 was also used to produce a cDNA library from urediniospores germinated over water, library designation PTDG, from which bi-directional reads were generated.

### Compilation of EST sequences and Pt gene finding

Table 1 summarizes the number of ESTs and derived unigene sets generated from the aeciospore, pycniospore, dormant teliospore, isolated haustoria and germinated urediniospore libraries. The 17,684 newly-generated sequences were pooled with 25,558 ESTs from our previous study [14] and 81 previously reported [13] to generate a non-redundant unigene set of 13,328 sequences (see Methods). The current study contributes 3,990 new, non-redundant unigene sequences to the database.

**Table 1:** cDNA libraries produced and number of generated quality EST reads

cDNA lib	source	designation ^a^	# reads	unigenes ^b^	singlets	contigs	predicted fungal ^c^	stage-specific ^d^
PT029	aeciospores	pte	1292	208 (16%)	166	42	199 (95%)	100 (48%)
PT030	pycniospores	ptp	4869	1252 (26%)	807	445	1116 (89%)	961 (77%)
PT031	dormant teliospores	ptt	3703	1165 (31%)	878	287	1041 (89%)	801 (69%)
PT033	isolated haustoria	ptih	4896	2886 (59%)	2620	266	307 (11%)	441 (15%)
PTDH	isolated haustoria	ptih	n/a	1921	354	1567	1236 (64%)	1069 (56%)
PTDG	germinated urediniospores	ptg	n/a	1003	83	920	740 (74%)	398 (40%)

^a ^life cycle stage-specific suffix, used at the end of all EST sequences: pte, *Puccinia triticina *aeciospores; ptp, pycniospores; ptt, teliospores; ptih, isolated haustoria; ptg, germinated urediniospores.

^b ^non redundant sequences obtained via CAP3 analysis of life cycle stage-specific EST set (as percent of reads generated), yielding singlets and contigs

^c ^number of unigenes predicted to be fungal (as percent of compiled unigenes for that stage)

^d ^number of unigenes not represented in other stages (as percent of all unigenes from this library)

Several of the cDNA libraries were generated from RNA isolated from infected plants and would contain host cDNA; other (bacterial) contaminants are also fairly abundant when such approaches are used. Therefore, we predicted the putative fungal genes using two approaches, *ab initio *and comparative genomics; we compared the results and now present a consolidated prediction set of 6,308 genes.

### Ab initio gene prediction approach using the EST version of GeneMarkS

**Figure 1:**
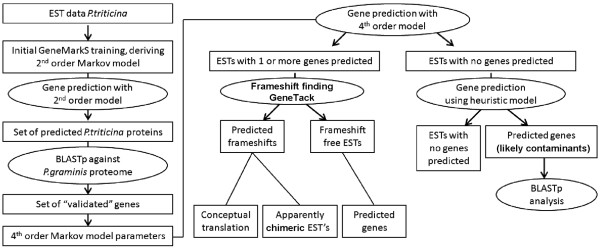
**Flow chart of *Pt *EST sequence analysis**. Gene prediction in the *P. triticina *EST sequences was done by the EST version of the GeneMarkS program [25]. The initial 2^nd ^order model of protein coding regions were derived automatically; then from the set of predicted genes we selected a reduced set of those that were likely to be fungal genes (see text) and more specific 4^th ^order model for fungal genes was derived. Frameshift detection in ESTs with one or more genes predicted in the same strand were analyzed by the frameshift prediction program GeneTack [28] (see text).

The EST version of GeneMarkS, an extension of a self-training method for gene calling [25], was used to derive gene models in three steps (Figure 1). First, from 13,328 *Pt *unigenes, we selected 10,576 sequences longer than 300 nt. This set of sequences was used as an input to GeneMarkS self-training to obtain an initial estimation of the parameters for the underlying generalized hidden Markov model (gHMM), particularly parameters of the 2^nd ^order Markov chain model of a protein-coding region. Thus, estimated parameters were used in GeneMark.hmm [26] to generate the set of *Pt *gene predictions. Notably, due to a likely presence of contaminant sequences, the initial set of genes may include genes from species other than *Pt *and therefore the set of parameters might be biased.

Second, to further improve estimates of the HMM parameters, we proceeded with validation of the automatically derived training set. We used BLASTP to search for homologs of predicted *Pt *proteins among 20,567 proteins encoded in the genome of *Pgt*. At a cut-off e-value of 1e^-10^, this procedure identified a set of 2,093 *Pt *genes with *Pgt *homologs. This reduced set was used as a refined *Pt *training set to derive parameters for the 4^th ^order Markov model of a protein-coding region, a critically important submodel of the gHMM.

**Figure 2:**
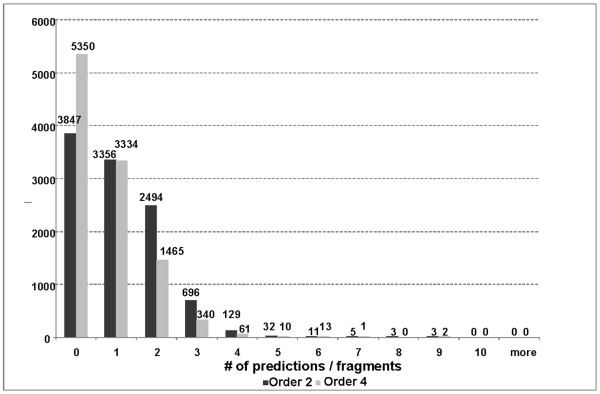
**Estimating *Pt *gene prediction in the EST unigene set**. Distribution of numbers of EST/unigene sequences with 0, 1, 2 ... genes predicted by the 2^nd ^order and the 4^th ^order Markov model.

Third, the improved gHMM was used to make gene predictions in the same 10,576 *Pt *unigene sequences as in step 1. Now a much smaller set of 7,681 predicted *Pt *genes was produced. Figure 2 shows the comparison between the results of analysis of EST fragments by the 2^nd ^and 4^th ^order models in terms of distribution of numbers of predicted coding regions per single EST fragment.

The 4^th ^order model was more specific; it omitted 3,579 genes identified by the 2^nd ^order model. A comparative G + C content analysis of the genes predicted by the 2^nd ^and 4^th ^order models showed that the genes predicted by the 4^th ^order model had a slightly higher G + C content than those predicted by the 2^nd ^order model (51% versus 48%). Notably, a number of genes now absent from the predictions could be attributed to contaminant sequences, possibly from the wheat host (see Contaminants section below).

Also, we observed that the use of the 4^th ^order model led to a significant increase, by 1,503, in the number of fragments with no genes predicted. A total of 5,350 unigenes out of 10,576 analyzed unigenes longer than 300 nt, were identified as containing no fungal genes. Still, these 5,350 unigenes did contain protein-coding sequences. When we switched gene prediction model in GeneMark.hmm to a generic 2^nd ^order heuristic model specific for genomic DNA with a given G + C content [27], we identified 5,156 protein coding regions (Additional file 1). However, at e < 10^-5^, BLASTP searches revealed similarity to *Pgt *proteins for only 14 out of these 5,156 predicted proteins and 19 had similarity to proteins in the NCBI nr database. Of the 19 "non-*Pgt*" proteins, at least 9 had similarity to proteins in plants, human and *E. coli *thus indicating possible contaminants (Additional file 2). The majority of the 5,156 predicted protein coding regions (3,501 or 68%) had lengths of less than 150 nt. It is likely that these EST sequences represent 5', or 3'- UTRs and predicted short coding regions are mere artifacts or represent very small proteins.

### Comparative genomics approach using BLASTN

As part of a *Pt *genome sequencing project (Cuomo, Fellers, Szabo and Bakkeren), an initial set of short genome sequences generated through '454 pyrosequencing' (see Methods) was available. The 13,328 EST unigene sequences were compared by BLASTN to this genome collection and 4,749 yielded significant matches at e < 10^-5^. This number differed from the *Pt *unigene set of 5,007 that we arrived at upon using GeneMarkS; a BLASTN comparison showed the two sets had 3,448 unigenes in common. At this early stage of *Pt *genome analysis it is uncertain how much coverage has been obtained with the 454-genomic reads. In addition, 13 out of the 14 unigene sequences not called by GeneMark.hmm but matching *Pgt *proteins by BLAST (see above), had BLASTN hits to the *Pt *genomic reads. We therefore assembled a final set of 6,308 putative *Pt *unigenes composed of the union of the two predicted sets. Of these, 3,383 (54%) were contributed through this new study and constitute over 85% of the total 3,990 newly-added unigene sequences. The percentage of called fungal sequences was even higher for individual spore stages (89 - 95%; Table 1) indicating the expected purity of collected spore samples.

Using the BLASTN algorithm, out of the total set of 13,328 EST unigenes, 6,095 did not match any sequences in the surveyed databases although 1,452 still matched the preliminary *Pt *genome reads in a BLASTN search and 1,656 the more-inclusive predicted fungal unigene set. Whether these sequences represent *Pt*-specific gene sequences or transcribed genome regions where no genes have (yet) been predicted, remains to be investigated. Although such sequences do not currently contribute to gene discovery, they will be valuable for the verification of gene models or transcribed regions in the completed genome sequence.

### Frameshift detection

Although one would expect to see just one continuous protein-coding region per unigene, in the output of the EST version of GeneMarkS with the *Pt*-specific 4^th ^order model, we could identify up to nine predicted genes per unigene and sometimes no genes (Figure 2). Multiple predictions could be explained by the presence of sequencing errors. To identify possible frameshifts caused by sequencing errors or chimeric cDNA inserts, a new frameshift prediction program GeneTack [28] was applied to 3,851 ESTs with several genes predicted in the same strand. Separate genes with no detected frameshifts were identified in 2,645 ESTs (69%) whereas a single frameshift was predicted in 905 ESTs (24%). Protein-coding regions with several frameshifts were identified in the remaining 301 ESTs (Additional file 3). Coding sequences with a single predicted frameshift were conceptually translated. This approach produced 805 sequences used to validate the GeneTack frameshift predictions by a BLASTP search against the *Pgt *proteome (20,567 sequences) and the NCBI non-redundant database (8,924,078 sequences). At e < 10^-5^, database hits overlapping the sites of predicted frameshifts confirmed 325 frameshift predictions; in most cases the frameshift was caused by misinterpretation of single-pass reads. When inspection of sequencing electropherograms allowed, the corresponding EST sequences were corrected.

### Contaminants

Several cDNA libraries were constructed from host infection stages. In order to not discard any potential fungal sequences from our unigene set, we employed a rather conservative strategy which most likely left in the database a number of "contaminating" wheat host sequences. To further understand the discrepancy between the total set of 13,328 EST unigenes and the 6,308 predicted fungal unigenes, all unigenes were also compared by BLASTN to a large collection of available wheat EST sequences to identify possible host contaminants. Some 3,080 unigenes matched wheat ESTs at e < 10^-5 ^and just over 3,300 had similarity to a 1x wheat genome coverage of '454-generated' sequences (http://www.cerealsdb.uk.net/index.htm). Still, 42% of unigenes matching wheat sequences (1,392 unigenes) also matched the preliminary *Pt *genomic reads. Since e-values are related to the size of the database, care has to be taken when directly comparing values obtained from databases of significantly different sizes. Nevertheless, in a smaller EST unigene set [14], we had also observed unigenes that matched fungal and wheat sequences with e-values in a similar range. Whether possibly horizontal gene transfer during co-evolution is involved might be solved once the genomes of both organisms are available.

Some 7,020 sequences were not included in the 'putative *Pt *fungal' set; they did not match the preliminary, still partial *Pt *genome reads. Of these 63% (4,439 unigenes) did not match any sequences in the databases surveyed. However, 26 sequences matched *Pgt *proteins at e < 10^-5^, 2% matched sequences in the nr database, 11% matched wheat ESTs, 22% matched fungal and oomycete ESTs in COGEME [29], and 30% matched sequences in EST-other. Those sequences without database matches (4,439 unigenes or 63%) were mainly derived from one appressorial library ('pta': 1,727 unigenes), from one specific germinated urediniospore stage library PT002 ('ptg': 1,598 unigenes), and from a wheat-infection stage library library PT009 ('pth': 520 unigenes; [14]). What these three cDNA libraries had in common was that cDNA inserts were generated by PCR which can reduce tag size. When the size distribution of these 4,439 unigene sequences was evaluated, almost 45% had lengths of less than 500 bp (compared to less than 29% among all 13,328 unigenes). When a comprehensive *Pt *genome is available, these sequences will be re-evaluated.

### Annotation and classification of ESTs

The 13,328 *Pt *EST unigene sequences and the predicted fungal subset were compared by TBLASTX or BLASTX to various databases. Using a cut-off value of e < 10^-5^, approximately 40% of the putative fungal subset of 6,308 unigenes matched NCBI's nr, with a similar number matching the UniProt database. The corresponding percentages were 27% for the fungal and oomycete-specific EST database COGEME and almost 60% for dbEST (Additional file 4). When the total 13,328-member unigene set was compared to dbEST, roughly a third more (2,147 unigenes for a total of 5,854 or 44%) matched, but most of these (1,628 unigenes) scored with low significance at e < 10^-10^.

Preliminary annotation was achieved for 2,556 unigenes by choosing the most informative header arising from the various database BLAST searches (at e < 10^-5^) which was not necessarily the most statistically significant return; 2,286 (90%) were judged to be of fungal origin and 48 had similarity to ribosomal sequences of various rusts (Additional file 5). It is notoriously difficult to assign genes to categories since the proteins they encode can have multiple functions and/or be associated with various aspects of their biology. However, to achieve a better understanding of their role(s), we analysed all unigenes with the program 'annot8r' [30] which assigned various GO (Gene Ontology), EC (Enzyme Commission number) and/or KEGG (Kyoto Encyclopedia of Genes and Genomes) annotations to almost 1,500 unigenes.

### Similarity searches to related fungi

To investigate relatedness of *Pt *sequences to those of its closest known related rust fungus for which substantial genomic resources have been developed, the initial 13,328-member *Pt *EST set as well as the 6,308-member predicted fungal set were compared to various sets of *Pgt *sequences using several algorithms. The set of 6,308 putative *Pt *unigenes matched fairly closely the numbers obtained for the initial 13,328 unigenes, especially for more conserved sequences (Table 2, 2^nd ^and 3^rd ^column), indicating that the *Pt *gene finding using the *Pgt*-trained GeneMark.hmm algorithm and the *Pt *genome filter, had indeed generated a *Pt*-enriched set. The discrepancy was larger when comparing to *Pgt *ESTs which could indicate that the available *Pgt *genome is not completely covered and/or, more likely, the presence of (host) contaminants in the available *Pgt *ESTs, such as the haustorial-specific set which has not been sanitized for fungal sequences. When predicted protein sequences from the *Pt *ESTs obtained through the BLASTX algorithm were compared to the predicted proteome from *Pgt *(Table 2, 4^th ^column), the numbers were somewhat lower than when compared to the complete translated *Pgt *genome using TBLASTX. This could indicate that the predicted *Pgt *proteome is a somewhat conservative underestimate or that there are more conserved genomic regions between the species that yield RNA and are therefore transcribed but not necessarily translated or might not be recognized by current computer models as *bona fide *ORFs. For example, small secreted proteins are often missed. The data in the TBLASTX column reveal on average more matches than those in the BLASTN column, especially in the higher-confidence intervals, which suggests more conservation at the protein level between species and might be expected from protein-coding cDNAs (ESTs). Of the 6,308 predicted fungal unigenes, BLASTX results indicated 2,550 (40%) had potential orthologs among the predicted *Pgt *proteins (at e < 10^-5^); among 4,749 unigenes that were identified through BLASTN matches with *Pt *genomic resources, this percentage was 55%.

**Table 2:** Similarity searches of *Pt *unigene sequences to related fungal databases

	*Pgt *ESTs *Pgt *genome ^a^		*Pgt *proteins ^b^	*Pt *genome ^c^	*Pst *ESTs ^d^		*Mlp *proteins ^e^	*U. maydis *ESTs ^f^	*U. maydis *genome ^g^
**e_value**	**BLASTN**	**TBLASTX**	**BLASTX**	**BLASTN**	**BLASTN**	**TBLASTX**	**BLASTX**	**TBLASTX**	**TBLASTX**

< = -100	863 (867) 131 (131)	490 (490) 394 (394)	285 (285)	(3426)	120 (120)	79 (70)	117 (117)	68 (68)	67 (67)
-100 < × ≤ -50	434 (442) 466 (466)	749 (751) 829 (829)	763 (763)	(678)	151 (151)	253 (254)	434 (434)	277 (277)	249 (249)
-50 < × ≤ -20	452 (481) 879 (879)	786 (815) 971 (972)	957 (958)	(395)	211 (213)	377 (381)	795 (796)	533 (535)	487 (489)
-20 < × ≤ -5	513 (612) 878 (878)	753 (849) 832 (899)	745 (764)	(250)	270 (292)	473 (516)	758 (781)	629 (656)	656 (680)
TOTAL	2262 (2402) 2354 (2354)	2778 (2905) 3026 (3094)	2750 (2770)	(4749)	752 (776)	1182 (1230)	2104 (2128)	1507 (1535)	1459 (1485)

^a ^the 6,308 most-likely *Pt *unigene subset (as well as the complete 13,328 *Pt *unigene sequences: numbers in parentheses) were compared to available *Pgt *ESTs and genome sequences (upper and lower line in each cell, respectively), using the algorithms indicated

^b ^results comparing the 20,567 and 18,241 (excluding mostly TE-related) gene calls, were identical

^c ^early *Pt *genome Sanger and 454 sequences

^d ^set of 3,297 published, most-likely *P. striiformis *f. sp. *tritici *EST sequences from NCBI dbEST [17,20,21]

^e ^set of 16,831 predicted proteins from the *Melampsora laricis-populina *genome (http://genome.jgi-psf.org/Mellp1/Mellp1.home.html)

^f ^predicted/valid transcripts (many matching ESTs, [43,56,57], Morrison *et al*. in preparation] and 364 additional EST unigenes not predicted in the *Um *genome at MIPS

^g ^MUMDB (http://mips.helmholtz-muenchen.de/genre/proj/ustilago)

The *Pt *unigene set was also compared to a set of 3,297 published, putative wheat stripe rust (*Pst*) ESTs from comparable life cycle stages (urediniospores and haustoria, Table 2). At e < 10^-5^, roughly 20% of the *Pt *fungal unigenes matched this *Pst *collection, whereas almost half matched the *Pgt *resources (see above) which themselves have an estimated genome coverage of approximately 92%. The current number of gene calls for *Pgt *is 20,567, 18,241 when disregarding many transposable element- (TE) related calls, and will likely be similar for *Pt *(and *Pst*). We have only compared a partial *Pt *gene set, based on the EST unigenes, but, assuming we can extrapolate the numbers, these surprisingly low percentages suggest substantial species-specific gene complements and could indicate considerable genome evolution. Indeed, even though these rust fungi are considered to belong to the same genus, they fall into well-separated phylogenetic sub-clades [31]. Gene divergence could occur due to strong selection of these biotrophs. By comparison, two related biotrophic smut fungi from different genera, *U. maydis *and *Sporisorium reilianum*, still share an average 74% amino acid identity among predicted proteins [32]. A closer look at the set of unigenes that matched *Pt *genome sequences but not *Pgt *resources (2,157 unigenes or 34%), revealed that 725 unigenes had representation in the '*in planta *infection' and 'isolated haustoria' cDNA libraries (out of a pool of 1,344 generated unigenes matching *Pt *genomic sequences), 719 in urediniospores (out of 1,627), 374 in pycniospores (out of 1,101), 247 in teliospores (out of 790) and less than 100 in other stages. As a percentage of their respective pools (54%, 44%, 34% and 31%), this suggests that genes expressed in these four life cycle stages might be under different selection pressures. Using this rough estimate, the highest discrepancy between *Pt *and *Pgt *is seen among genes expressed during plant infection and it could be argued that many of those represent virulence factors likely under selection pressure as has been seen among poplar rust, *Melampsora *species [33].

A particular focus of this study was to compare the *Pt *unigenes to *U. maydis *genomic resources since this fungus is the best-studied, closest-related model representing cereal-biotrophic basidiomycete interactions. We have previously illustrated the possibility of using the *U. maydis *model system for performing functional analyses of *Pt *genes [34]. A BLASTX search against the 6,846 predicted *U. maydis *proteins [35] yielded 1,535 matches at e < 10^-5^, 907 (13% of the total *Um *complement) with significant similarity at e < 10^-20 ^(Additional file 6). Most matching *Pt *unigenes (1,506) were predicted 'fungal' as might be expected from genes having orthologs in basidiomycetes belonging to different genera (Table 2). Indeed, a large majority of 1,416 *Pt-Um *"pairs" (92%) also matched *Pgt *proteins suggesting the existence of a common set of proteins/functions among these basidiomycetes. However, 119 *Pt-Um *"pairs" (8%) did not match *Pgt *proteins. In a complementary approach, the complete set of 20,567 predicted *Pgt *proteins was compared to the same set of *Um *proteins, yielding 5,624 matches of which 4,012 (59% of the total *Um *complement and 20% of the *Pgt *complement) revealed significant similarities at e < 10^-20^. Of these *Pgt-Um *"pairs", 2,604 (46%) also matched *Pt *unigenes; this smaller percentage likely reflects the large comprehensive *Pgt *genome-predicted protein set compared to the *Pt *EST subset. The identification of many homologs and the existence of many *Um *gene deletion mutants or the ease with which *Um *gene deletions can be obtained [36], could allow for functional *Pt *gene analyses through complementation in *U. maydis *[34].

### Spore-specific unigenes

**Figure 3:**
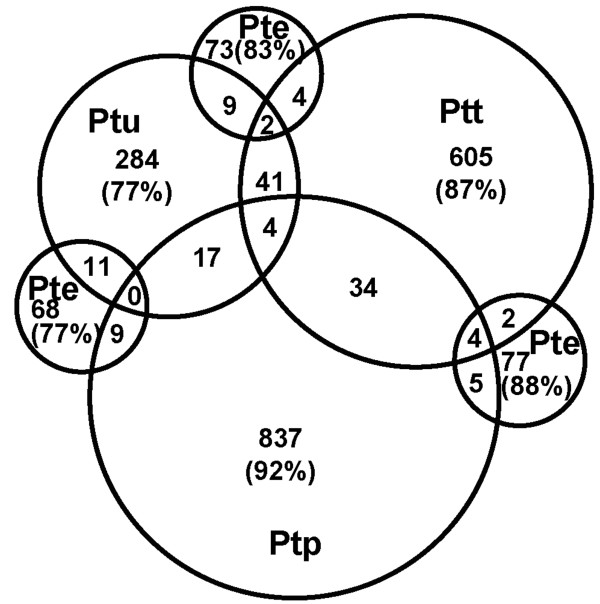
**Venn diagram depicting numbers of predicted putative fungal *Pt *unigenes uniquely found in and shared among spore stages**. The sizes of the circles reflect the pool size of unigenes for each stage: 369 Ptu (urediniospores), 697 Ptt (teliospores), 88 Pte (aeciospores) and 911 Ptp (pycniospores). One unigene was shared by all spore stages. All numbers reflect counts in that particular section only.

One unique aspect of this study is the generation of ESTs from sexual spores from this macrocyclic, heterocious cereal rust. Teliospores, pycniospores, aeciospores and urediniospores were fresh and considered to be in a 'dormant' stage ready for dispersal, although the physiologic state of pycniospores in nectar is uncertain. The non-redundant unigenes generated for each spore type were filtered for putative fungal sequences (Table 1) and the overlap between spore-specific transcripts was assessed (Figure 3). Although it is unlikely that we sampled the cDNA libraries to saturation, it was striking that all four spore stages generated a large percentage of seemingly unique sequences. RNA sequencing or microarray analyses will have to be performed to verify levels of transcription, but the data presented here suggests that these unique stages express sets of genes that are needed for specific developmental programs. Tentative annotations were assigned to 394 unigenes and are given in Additional file 7. Overall, each spore stage seemed to express large numbers of genes involved in metabolism, energy production and conversion, translation and protein turn-over (ubiquitin-related). Pycnio- and teliospores seemed to have yielded a larger number of ribosomal protein genes possibly related to more active protein production. We noted also several transporters (such as a hexose transporter in pycniospores, a homolog of which was also identified in haustoria) and several possible transcription factors, such as seven ring- or Zn-finger containing proteins in pycniospores. Interestingly, the urediniospore stage revealed the best annotated set relative to the number of unigenes, followed by pycniospores, then teliospores and a disproportionate set was found for aeciospores. Whether this reflects larger sets of novel genes expressed in these latter stages, remains to be discovered.

Several unigenes revealed interesting putative functions, in particular among the ESTs from the pycniospore library, a stage not yet covered in the literature. PtContig7448 matched a pheromone transporter from *Cryptococcus neoformans*, a human opportunistic basidiomycete pathogen, and was covered by sequences from 4 different cDNA clones from only that cell type. Contig7830 (one cDNA clone) matched pheromone receptor sequences in many fungi (e.g., the A2 pheromone receptor from *Microbotryum violaceum*, the anther smut fungus) and was similar to a 357-amino acid protein in *Pgt*. *Pt *and related rusts are thought to possess bipolar mating systems (+ or -) [37] although some might possess multiple-allelomorphic tetrapolar systems similar to the higher mushrooms [38]. Because pycniospores are haploid cells in which mating type has segregated and are primed for mating to form the dikaryotic aeciospores, they are expected to express mating-type specific genes. In rusts, no information on mating-type genes has been published. It would be tremendously valuable to uncover the molecular basis of the mating system and reveal the structure of *MAT *loci in cereal rusts. The revealed *Pt *pheromone receptor gene, *pra*, could represent a component of the mating-type locus. Contig7901 (one cDNA clone) has similarity to a protein belonging to the oligopeptide OPT transporter family found in many fungi and annotated as 'sexual differentiation process protein isp4'. The pycniospore stage also revealed several genes involved in sterol biosynthesis with possible roles in mating (Contigs 7279, 7412, 7754 and 7777). In *S. cerevisiae*, (ergo-)sterols have been shown to be involved in cell fusion during mating, for example, sphingolipid and ergosterol biosynthetic mutants fail to polarize proteins to the tip of the deforming yeast cell ("shmoo") and are therefore deficient in mating [39,40]. However, another ergosterol biosynthesis gene, Erg28 (Contig7716) and an oxysterol-binding protein (PT0318.P01.C21.ptt), were found in the teliospores library, whereas a sterol O-acyltransferase (Contig107) had one representative EST in urediniospores and one in germinated urediniospores. Among the whole unigene set, a few Contigs, 7490 (2 cDNA clones), 7853 (9 cDNA clones) and 7905 (2 cDNA clones), were obtained only from the pycniospore library and were most similar to *Pgt *predicted protein PGTG_01034.2, a carbon catabolite-derepressing protein kinase. A comparative multiple sequence alignment of DNA sequences in AlignX confirmed all three *Pt *unigenes were closely related but had four approximately 80 bp-stretches of DNA that were shared in different combinations. Whether these represent unique genes or are different splice products will be solved once the *Pt *genome is available; however, the unique stretches did not match predicted introns in various *Pgt *homologs of which there were many (at least 3 at e = 0). We are interested in developmental-specific kinases and intriguingly, these *Pt *contigs also bore great resemblance to the catalytic subunit of cAMP-dependent protein kinase, known to be involved in responsiveness to mating pheromone in several fungi [41,42].

Teliospores are survival propagules and are melanized for protection. Contig7966 (one teliospores-specific cDNA clone), matched a diphenol/urishiol (multi-copper) oxidase or laccase (EC:1.10.3.2), an enzyme found in fungi and plants which oxidizes different phenols and diamines and is implicated in the production of melanin pigment.

Several unigenes had EST representation mainly in all spore stages: Contig5605 (TAR1, regulation of mitochondrial gene expression), Contig8158 (a P450 monooxygenase) and Contigs 279, 5253 and 7316 (no hits). It is possible that certain genes are involved in general spore-forming processes despite their very different morphology and physiology.

### Correlating elevated EST levels of spore-specific libraries between P. triticina and U. maydis

Spore production is critical to the dispersal of rusts and smuts; therefore, knowledge of the development and biology of spores is essential to understanding disease spread. Since spore development in these fungi requires growth in the host plant, it is reasonable to assume that their development is triggered in response to a signal(s) received from the host. In this context, identifying genes with elevated transcript levels in spores of both fungi may lead to the discovery of common responses to plant signals. Of the four *Pt *rust spore stages we generated ESTs from in this study, only teliospores have a biological equivalent in the life cycle of *Um *(represented by ESTs from the 'dormant teliospore' cDNA library TDO [43]). Comparing expression among libraries of these two species is complicated by variation in library preparation methods, notably the requirement to include an amplification step to clone cDNAs from the low levels of *Pt *teliospores, and the need to normalize libraries being compared to correct for EST pool size. With these limitations, we expected to find few commonly expressed genes. Additional file 6 shows that *Pt *contig5874, a putative mitochondrial inner membrane protein involved in protein import, has elevated numbers of ESTs in both teliospore derived libraries. Elevated numbers of ESTs in *Pt *contig7329, a NADH-ubiquinone oxidoreductase, originated from *Pt *pycniospore and teliospore libraries. The similar um10989 (e = 10^-34^) has elevated EST numbers in *Um *teliospore and haploid cell libraries (Additional file 6). *Pt *pycniospores and *Um *haploid cells are both descendents of basidiospores, the direct products of meiosis. The discovery of these genes is interesting and they deserve further investigation. However, the fact that we found any commonly expressed genes given the limitations in the analysis is perhaps more important and supports a deeper analysis of the teliospore transcriptome in these fungi.

Comparing libraries from other spore (cell) types that are functionally equivalent between *Pt *and *Um *revealed further similarities in elevated EST levels. Four *Pt *contigs with elevated numbers in the pycniospore had homologs with elevated numbers in *Um *haploid cells or germinating teliospores. Two represent conserved hypothetical proteins and the others represent cofilin, an actin binding and severing protein, and a 26S proteasome non-ATPase regulatory subunit (Additional file 8). Six *Pt *contigs having elevated EST counts in the germinated urediniospore stage had *Um *homologs with higher number of ESTs, relative to the other stages, in the functionally equivalent *Um *dikaryon (Additional file 8). It is notable that four of these were conserved hypothetical proteins. These may represent functionally conserved genes. The function of these genes can be investigated in the tractable *Um*. Further expression analysis is required to confirm these results, but finding similarly high EST counts between similar cell types supports the benefit of continuing these comparative approaches in more depth using RNA sequencing techniques.

### Stage-specific unigenes

Figure 3 presented the overlap between predicted fungal, spore-specific *Pt *unigenes. In Table 3, an inventory is presented of the number of unigenes found uniquely in these spore stages as well as during urediniospore germination and in isolated haustoria. When numbers per stage are extrapolated relative to the largest number of reads generated (represented by the ptg stage) as to mimic equal sampling, pycniospores, teliospores and isolated haustoria would yield the largest sets of unique gene sequences with most in the latter. The smallest and equal sets were found in aeciospores and germinating urediniospores; possibly, more common genes are expressed in these stages. It can be argued that the function of aeciospores and germinating urediniospores is the same: infecting wheat. Interestingly, the largest set of unique unigenes with the least similarity to *Pgt *predicted genes was found in isolated haustoria. This comparison strengthens the observation made earlier, that haustoria likely express stage- and species-specific genes, possibly representing virulence factors among which could be effectors that have been under diversifying selection [33].

**Table 3:** Stage-specific *Pt *unigene counts and representation in *Pgt*

stage	# unique unigenes ^a^	predicted fungal ^b^		# matching *Pgt *homologs ^d^
		**counts**	**normalized ^c^**	

ptp	961	911	2246	563 (62%)
ptt	801	697	2259	374 (54%)
pte	100	88	818	47 (54%)
ptu	486	369	1131	152 (41%)
ptg	3308	**811**	**811**	475 (59%)
ptih	1283	973	2430	292 (30%)

^a ^number of non-redundant sequences obtained from the indicated specific developmental stage only: ptp, pycniospores; ptt, teliospores; pte, aeciospores; ptu, dormant urediniospores; ptg, germinated urediniospores; ptih, isolated haustoria

^b ^number of unigenes predicted to be fungal

^c ^number of predicted fungal unigenes for that stage was normalized using quality reads in relation to the stage with the highest sampling, ptg (bold type)

^d ^BLASTX search of Pgt genome-predicted proteins at e < 10^-5 ^(percent of actual predicted fungal unigenes)

### Secretome-specific unigenes

The combined 13,328 unigene set was queried using InterProScan to identify protein sequences predicted to be secreted. To complement this, 11,638 proteins predicted from the partial *Pt *genome were queried using the method by Joly *et al*. [33] to yield 758 potentially secreted *Pt *proteins. Reciprocal BLAST searches were performed to generate a list of *Pt*-specific, potentially secreted proteins. This list was complemented by searches against a subset of 1,699 potentially secreted *Pgt *proteins derived similarly from the 20,567 genome-predicted *Pgt *proteins, and additionally against collected sets of various predicted "secretome" proteins: 689 assembled EST sequences from various poplar leaf rust fungi, *Melampsora *spp. (S + category in [33]), 28 identified flax rust, *Melampsora lini *haustoria-specific secreted proteins [23], 100 bean rust, *Uromyces fabae*, secretome proteins [44], and 386 described secreted proteins in *U. maydis *[45]. Additional file 9 shows which *Pt *unigenes are predicted to be secreted or are similar by BLAST searches to predicted secreted proteins in other (related) rust or basidiomycete plant biotrophic fungi. The representation of the ESTs among the various cDNA libraries is indicated, representing expression in the respective life cycle stages. Striking were the large groupings revealed; a given *Pt *unigene would match various different sequences, up to 27 *Pgt *proteins in the case of Contig75, with various e-values. These are likely family members of related secreted proteins, some of which seem to belong to clusters (such as PGTG_8431 to PGTG_8436, and PGTG_04883 to PGTG04887). Although many such groups were found matching *Pgt*, large poplar rust groups were also evident, and often a *Pt *unigene would match similar large groups in both organisms; sometimes they would match uniquely in poplar rust. A TBLASTX search of 583 *Pt *unigenes predicted to be secreted (based on the searches mentioned above) against the complete *Pt *unigene set ('self-BLAST') yielded related *Pt *groups which in general correlated well with the *Pgt *and poplar rust groups (Additional file 9, 'Pt secr prots self-BLAST' tab).

**Figure 4:**
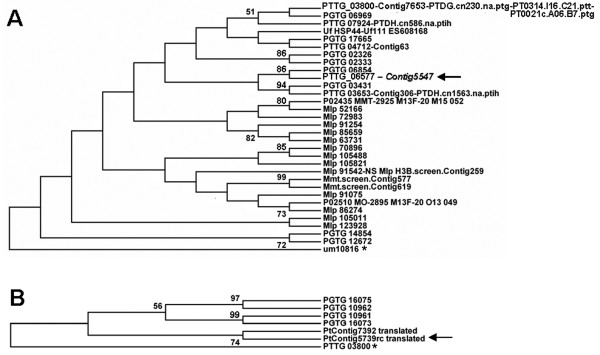
**Molecular relationships among some families of SSPs expressed during infection**. (A) 31 related predicted SSPs common to four rust fungal species. Comparisons were seeded by *Pt *Contig5547 (arrow) which represents a gene expressed and, based on EST counts, possibly induced upon infection. *Pt *proteins predicted from the genome representing the unigenes were used in the comparison; several "unigenes" represented the same *Pt *protein indicating they did not collapse into one contig for various reasons (possibly representing allelic sequences). (B) Relationship of a small family of *Puccinia*-specific, small (~118 aa) SSPs; the two *Pgt *pairs are likely allelic proteins. Note that no proper gene calls exist (yet) for the *Pt *proteins and thus more members could exist. Relationships based on a ClustalW alignment (Additional file 10) were inferred using the Maximum Parsimony method (Close-Neighbor-Interchange algorithm, gaps and missing data were eliminated from the dataset). Bootstrap consensus tree (1000 replicates) with values at the nodes. SSPs indicated with an asterisk were used as outgroups. Phylogenetic analyses were conducted in MEGA4 [55].

In general, larger, expanded families tended to have related sequences in all other rusts, including the bean rust (which is only represented by a small partial EST set). This suggests that such families originate from a more-conserved protein(s) in a common ancestor and likely evolved paralogous family members in the various species. Among the *Pt *unigenes coding for SSPs that seemed to be *Puccinia*-specific (*Pt *and *Pgt*), the families tended to be smaller. Figure 4A illustrates an example of a family of proteins that is rather conserved among the various rusts, including the bean rust. We started from *Pt *contig5547, a tentative metallothionein, represented by 5 ESTs from the haustorial stage, with 13 matches in poplar rust and 9 in stem rust. In this comparison, small clades contained mainly paralogous *Puccinia *or *Melampsora *proteins, but there was an overall similarity suggesting the existence of an ancient 'founding' gene member. Figure 4B shows an example of *Puccinia*-specific SSPs that likely evolved paralogs after speciation. This preliminary *Pt *unigene data set merits close scrutiny on a molecular phylogenetic level once all genomes have been completed and proper protein calls are available.

Overall, 2,421 predicted fungal unigenes, of which 1,344 matched *Pt *genomic 454 reads, had at least one EST generated from plant-infected material, represented by the stages pth, pti or ptih. Of the 1,402 unigenes representing isolated haustoria, 337 did not match any sequence in various databases including *Pgt *proteins, but of these, still 196 were predicted to be fungal and 157 matched *Pt *genomic reads. These could represent species-specific genes that are potentially preferentially expressed in haustoria, among which could be effectors. Transcript profiling or gene-specific quantitative PCR will be needed to verify whether these genes are expressed uniquely or at elevated levels during plant infection and hence might play important roles.

## Conclusions

The *Pt *EST sequences generated, collected and analysed in the current study, enriched by sampling spore stages not covered in other studies, will provide essential information for proper gene calling and annotation of the *Pt *genome currently being assembled. For the first time, all spore stages from a macrocyclic cereal rust fungus, except for basidiospores, have been investigated for expressed genes through this EST approach. Many obtained unigene sequences from the various spore stages seemed specific suggesting radically different developmental gene sets are involved. Unfortunately, many could not be annotated due to lack of similar sequences in the various databases. Comparing potential spore-specific gene sequences between *Pt *and the model basidiomycete plant biotroph *U. maydis*, seems feasible and has yielded some interesting candidates for follow-up studies.

Although substantial homology exists between the *Pt *unigene sequences and near-comprehensive genomic resources from the related stem rust fungus, *P. graminis *f. sp. *tritici*, many genes seem to have diverged substantially from those from the putative common ancestor and many others appear to be species specific. A preliminary comparative sequence analysis of several BAC clones harbouring large *Pt *genomic inserts with the syntenic regions in *Pgt*, seems to corroborate this (JF and GB, unpublished). Future comprehensive comparisons once the genomes of several cereal rust species are available will shed light on the molecular evolution of their genes and genomes.

The generated ESTs have also been invaluable in generating molecular markers. The current gene sequences have been used to reveal SSRs (Simple Sequence Repeats or microsatellites) which were used in genetic diversity studies [46], and the variation among compiled contigs, potentially being derived from two parental nuclei in case of the dikaryotic material or the 4 different isolates used, revealed SNPs (Single Nucleotide Polymorphisms) which are being used to saturate a *Pt *genetic map (unpublished).

The computational prediction of a comprehensive secretome for *Pt *is one of the goals once the genome sequence is completed. Many secreted proteins will function in general fungal development such as cell wall biogenesis, but a significant subset will be crucial to support the biotrophic life style and hence will be considered virulence factors. Some will function in generating and maintaining the matrix surrounding haustoria and possibly hyphae roaming the plant tissue and contribute to feeding, but others, effectors among them, will be needed to subdue the host defence machinery. It is anticipated that many of the effectors will represent the many avirulence gene products that trigger resistance in the numerous wheat cultivars. Unravelling this complement is crucial in revealing old and new interactions which will aid in breeding durable resistance in our cereal crops.

## Methods

### Fungal strains and plant materials

To produce teliospores, *Pt *race1 (BBBD; [47]) was inoculated on susceptible wheat (*Triticum aestivum *L.) cultivar Thatcher and the infection was allowed to proceed for months until senescence of the plants was induced by reducing watering and temperature. Dormant teliospores were then dissected from senescent wheat stalks with a fine scalpel; this material was used to generate cDNA library PT031. Aeciospores and pycniospores were collected from the alternate host, meadow rue or *Thalictrum speciosissimum*, infected with germinating and basidiospores-producing telial material (isolate 99193) in the greenhouse (Y. Anikster and T. Eilam). Pycnial nectar with spores was collected and dried in a lyophilizer for 24 h; this material was used to construct cDNA library PT030. Aeciospores were dissected from infected *T. speciosissimum *leaves resulting in cDNA library PT029. Haustoria were isolated from susceptible wheat, cv. Wichita, heavily-infected with *Pt *urediniospores from isolate PRTUS6 (race PBJL; [48]), using conA-loaded columns essentially as described [49]; this material generated cDNA library PT033 (J. Fellers). The latter method was also employed to isolate haustoria from wheat cv. Vuka heavily infected with isolate WBRS-97-3 (material for cDNA library PTDH, M. Dickinson). The same isolate was also used to generate urediniospores which were then germinated over water for 8 hours and then collected as described [14] yielding material for cDNA library PTDG (M. Dickinson).

### Isolation of total RNA and cDNA library construction

Dissected teliospores, haustorial preparations and germinated urediniospores were ground to a fine powder in liquid nitrogen using a mortar and pestle. The minute amounts of aeciospore and pycniospore material were ground in Eppendorf tubes with sterile sand. Total RNA was extracted in a solution of phenol-guanidine isothiocyanate (TRI Reagent, Molecular Research Center, Inc., Cincinnati, OH) according to instructions provided by the manufacturer. Due to the very small sample sizes, only 0.05 μg of total RNA could be obtained from the pycnio-, and aeciospores, and 1 μg from the teliospores. From these materials, cDNA libraries were constructed from total RNA using the SMART cDNA library kit (Clontech) according to the manufacturer's instructions with minor changes. The cycle number of LD PCR for cDNA synthesis was adapted; 26 cycles for pycnio- and teliospores, and 2 times 26 cycles (using an aliquot of the first amplification for the next 26 cycles) for aeciospores. The cDNA PCR products were digested with SfiI, then with proteinase K, and subsequently size-fractionated on 1% agarose gels in 0.5xTBE (45 mM Tris, pH 7.0, 45 mM boric acid, 1 mM EDTA). Fragments larger than 600 bp were excised and purified with the QiaQuick Gel Extraction Kit (Qiagen, Mississauga, ON). DNA fractions were ligated directionally into vector pDNR-LIB and transformed into *E. coli *DH10B/r. Total RNA from haustorial and germinated urediniospore material was converted to cDNA using oligo-dT primers containing a Xho1 site and Reverse Transcriptase (ZAP cDNA synthesis kit from Strategene which contains StrataScript™ Reverse Transcriptase). After adding EcoRI linkers to blunted 5'-ends, cDNA was cloned into vector lambda-zap; conversion of the library resulted in cDNA clones in pBluescript SK- in *E. coli *DH10B/r. RNA isolation and construction of cDNA library PT033 (isolated haustoria) was according to Li *et al*. [50].

### DNA sequencing

DNA sequencing of the cDNA clones from the teliospore -, aeciospore -, pycniospore - and isolated haustoria (PT033) stages was performed at the Michael Smith Genome Sciences Centre (Vancouver, BC, Canada). Clones were picked on a Genetix Q-Pix into 384-well plates containing 80 μL 2× YT medium with 7.5% glycerol and plasmids were isolated from 96-well plate cultures using a modified alkaline lysis method. Inserts representing the 3'-end of the mRNA were sequenced using the standard M13 forward primer (F: 5'-GTAAAACGACGGCCAG, or B21/C21: 5'-TGTAAAACGACGGCCAGT), the T7 promoter primer (B7/C7: 5'-AATACGACTCACTATAG) or primer TB (5'-(T)23 A/C/G) used to overcome stuttering and slippage during sequencing due to long polyT stretches. Inserts representing the 5'-end of the mRNA were sequenced using M13 reverse primers (BR/CR: 5'-CAGGAAACAGCTATGAC, or R/CPTR: 5'-AACAGCTATGACCATG). The BigDye Terminator Cycle Sequence chemistry was used for all reactions (Applied Biosystems, Foster City, CA). Sequences were run on the ABI3700 platform (Applied Biosystems). The isolated haustoria (PTDH) and germinated urediniospores (PTDG) cDNA sequences contributed by M. Dickinson were end-sequenced by Syngenta UK and run through the Stackpack program, which aligns identical sequences to give a non-redundant output. The sequences were also "trimmed" to remove any contaminating vector sequences that may interfere with database searching.

### ESTs database analysis

Generated ESTs were trimmed via the Consed suite of programs [51], including the 'Cross-Match' algorithm [52] using the UniVec database (http://www.ncbi.nlm.nih.gov/VecScreen/UniVec.html) to remove vector sequences and low quality reads. Sequences shorter than 50 bp were discarded and all were visually inspected. The resulting quality sequences, including the previously generated *Pt *ESTs [14] and a small set of unigene sequences from infected wheat material predicted to be fungal [13], were assembled into a non-redundant unigene set via the CAP3 assembly program [53]. Databases for the various comparisons were comprised of publicly available and assembled sequences from wheat where care was taken to include only sequences derived from "clean", uninfected material, and from various fungi and oomycetes such as the COGEME db, http://cogeme.ex.ac.uk/[54]. More specific collections were from the closely related *Pgt *genomic resources, such as the 20,567 predicted protein sequences or the 18,241 when disregarding many TE-related protein calls (release Jan 2010) and over 80,000 ESTs [19]; http://www.broadinstitute.org/annotation/genome/puccinia_group/MultiHome.html), as well as 30,000 *Pt *genomic BAC clone end-reads and initial pyrosequencing (454)-generated *Pt *genome fragments (http://www.ebi.ac.uk/ena/data/view/Taxon:208348). A collection of 3,297 putative fungal *Pst *EST sequences was collected from NCBI dbEST [17,20,21]. *Mlp *genomic resources were collected from JGI (http://genome.jgi-psf.org/Mellp1/Mellp1.home.html). *U. maydis *genomic resources were retrieved from the Munich Information Center for Protein Sequences (MIPS), MUMDB (http://mips.helmholtz-muenchen.de/genre/proj/ustilago).

### Accession numbers

The 17,684 new *Pt *EST sequences presented in this article can be retrieved from dbEST at NCBI (http://www.ncbi.nlm.nih.gov/sites/entrez?db=nucest) under accession numbers GR487994 to GR505442 and GR911120 to GR911355. Contig (assembled) unigene sequences are available from the Transcriptome Shotgun Assembly Sequence Database, TSA, at NCBI (http://www.ncbi.nlm.nih.gov/Genbank/TSA.html). Accession numbers are also given in Additional file 5.

## Authors' contributions

JX constructed the *Pt *telio-, aecio- and pycniospore cDNA libraries and performed some sequencing. RL compiled all comprehensive datasets and performed bioinformatic analyses. JF constructed an isolated haustoria cDNA library and edited the manuscript. MD constructed one of the isolated haustoria and the germinated spore cDNA libraries and provided the corresponding sequences. WZ performed adjustments to and all runs of GeneMarkS and subsequent analysis. IA performed frameshift prediction using the GeneTack program and further BLASTP validation. MB developed a strategy of analysis and edited the manuscript. TE and YA developed the techniques to induce teliospore formation and teliospore germination under controlled conditions to enable infection of the alternate host and obtain pycnia and aecia. BM provided some of the *Pt *fungal material and edited the manuscript. TB provided bioinformatic support on the analysis of *Pt *ESTs and generation of the database. SM and MM performed large-scale sequencing of the *Pt *clones at the GSC while BW and JA performed direct bioinformatic analysis of the *Pt *ESTs there; RM coordinated *Pt *sequencing at the GSC. DLJ performed bioinformatic analysis and comparisons with poplar rust species. BS developed the *Um *EST resources and was PI on an NSERC strategic grant that enabled the sequencing of subsets of *Pt *and *Um *ESTs; he also provided edits to the manuscript. MED and BS provided data on *Um *ESTs to enable the correlation of spore-specific expression between *Pt *and *Um*. GB was PI, conceived the project, supervised JX and RL, coordinated the joint contributions, analyzed the data and wrote the manuscript. All authors read and approved the final manuscript.

## Supplementary Material

Additional file 1 **Number of gene predictions on each *Pt *EST/unigene sequence in the set not previously called by the *Pgt*-trained 4**^**th **^**order model**. Click here for file

Additional file 2 **BLASTP analysis of predictions in "zero" set, against proteins predicted from the *Pgt *genome and the NCBI non-redundant database**. Click here for file

Additional file 3 **Statistics of frameshifts predicted by GeneTack in *Pt *ESTs with genes called in the same strand by GeneMarkS with the 4^th ^order model (supporting Figure 2)**. Click here for file

Additional file 4 **Similarity searches of *Pt *unigene sequences to various databases**. This table shows the number of unigenes matching the various databases within certain ranges of e-values. Click here for file

Additional file 5 **Annotation results**. This table holds the most-likely annotation of 2,551 unigenes based on searches against several databases. Source of the EST and number of independent sequences from various stages is indicated as are the unigene sequence match to the preliminary *Pt *genome 454 reads, the prediction by GeneMark and the closest homolog found among the predicted *Pgt *proteins. GO-annotations are also given according to cellular location, function or process, as well as EC and KEGG annotations (as produced by annot8r). Click here for file

Additional file 6 ***Pt *unigene sequences matching *U. maydis *ESTs**. The file shows possible homologs between the two species and their representation over the various cDNA libraries (life cycle stages). Click here for file

Additional file 7 ***Pt *non-redundant unigene sequences specific for and shared between spore stages as presented in the Venn diagram (supporting Figure 3) with annotation results**. Click here for file

Additional file 8 ***Pt *unigenes whose EST counts in the various spore stages correlate with EST counts of *U. maydis *homologs in equivalent cell types/spore stages**. Click here for file

Additional file 9 ***Pt *unigenes were compared to sets of known or predicted (small) secreted proteins (SSPs)**. This file reveals families of likely paralogous proteins and their matches to orthologous and homeologous predicted SSPs in various in rust fungi and *U. maydis*. It includes representation of ESTs over the sampled stages. Click here for file

Additional file 10 **ClustalW alignment of two families of predicted (small) secreted proteins (SSPs)**. The alignment was used for the construction of the phylograms in Figure 4. Click here for file
