# Supplementary material for: Gene discovery in EST sequences from the wheat leaf rust fungus Puccinia triticina sexual spores, asexual spores and haustoria, compared to other rust and corn smut fungi
Source: BMC Genomics. 2011 Mar 24;12:161. doi: 10.1186/1471-2164-12-161 (PMC3074555; doi:10.1186/1471-2164-12-161)
Supplement: Additional file 1 — Number of gene predictions on each Pt EST/unigene sequence in the set not previously called by the Pgt-trained 4th order model. [file 1471-2164-12-161-S1.DOC]

**Additional file 1: Number of gene predictions on each *Pt* EST/unigene sequence in the set not previously called by the *Pgt*-trained 4^th^ order model**

| **# of genes predicted** | **1** | **2** | **≥3** | **0** | **Total** |
| --- | --- | --- | --- | --- | --- |
| **# of unigene fragments** | **2299** | **1060** | **234** | **1757** | **5350** |
| **# of predictions** | **2299** | **2120** | **737** | **-** | **5156** |
| **# of predictions with length <150 nt** | **1436** | **1484** | **581** | **-** | **3501** |
