# Supplementary material for: Gene discovery in EST sequences from the wheat leaf rust fungus Puccinia triticina sexual spores, asexual spores and haustoria, compared to other rust and corn smut fungi
Source: BMC Genomics. 2011 Mar 24;12:161. doi: 10.1186/1471-2164-12-161 (PMC3074555; doi:10.1186/1471-2164-12-161)

Additional file 2. BLASTP analysis of prediction in "zero" set, against proteins of *P. graminis* genome


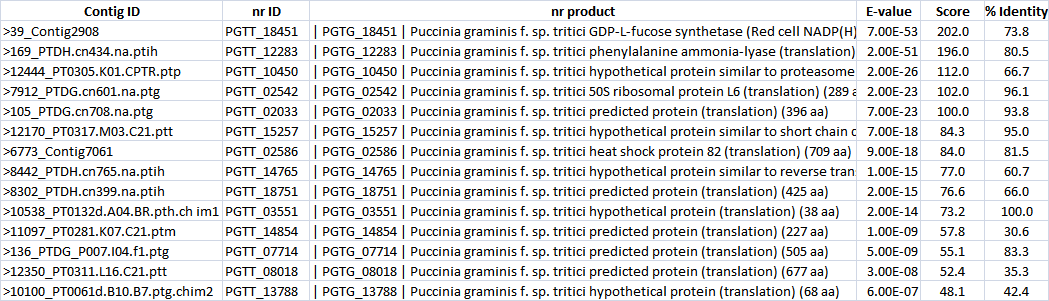


and NCBI non-redundant database.


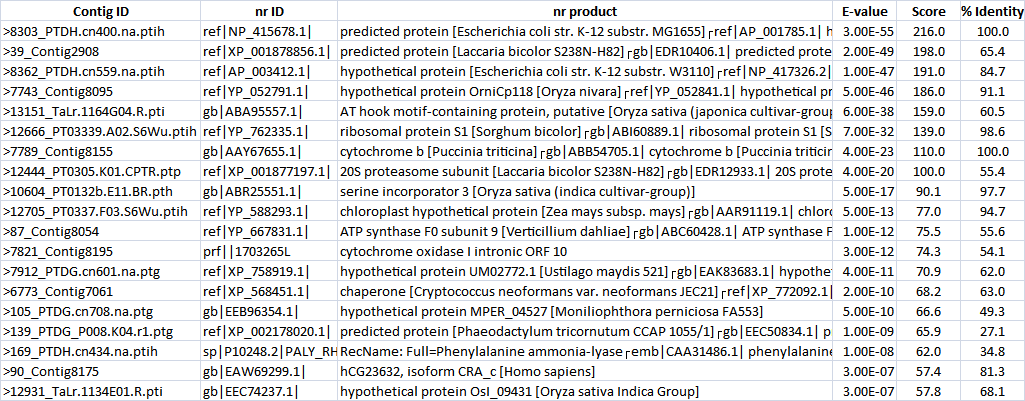

Supplement: Additional file 2 — BLASTP analysis of predictions in "zero" set, against proteins predicted from the Pgt genome and the NCBI non-redundant database. [file 1471-2164-12-161-S2.DOC]
