# Supplementary material for: Gene discovery in EST sequences from the wheat leaf rust fungus Puccinia triticina sexual spores, asexual spores and haustoria, compared to other rust and corn smut fungi
Source: BMC Genomics. 2011 Mar 24;12:161. doi: 10.1186/1471-2164-12-161 (PMC3074555; doi:10.1186/1471-2164-12-161)
Supplement: Additional file 3 — Statistics of frameshifts predicted by GeneTack in Pt ESTs with genes called in the same strand by GeneMarkS with the 4th order model (supporting Figure 2). [file 1471-2164-12-161-S3.DOC]

**Additional file 3. Statistics of frameshifts predicted by GeneTack in *Pt* ESTs with genes called in the same strand by GeneMarkS with the 4^th^ order model (see Fig. 1)**

| **Initial data** | | | **GeneTack results** | | |
| --- | --- | --- | --- | --- | --- |
| **# of predicted genes** | **Total # of ESTs** | **# ESTs with all genes located on the same strand** | **Total # of ESTs analyzed by GeneTack** | **# of ESTs with no frameshift predicted** | **# of ESTs with one frameshift predicted** |
| 1 | 3334 | 3334 | 3018 | 2302 | 588 |
| 2 | 1465 | 768 | 721 | 310 | 279 |
| 3 | 340 | 111 | 100 | 32 | 32 |
| 4 | 61 | 8 | 8 | 1 | 4 |
| 5 | 10 | 2 | 2 | 0 | 1 |
| 6 | 13 | 2 | 2 | 0 | 1 |
| 7 | 1 | 0 | 0 | 0 | 0 |
| 8 | 0 | 0 | 0 | 0 | 0 |
| 9 | 2 | 0 | 0 | 0 | 0 |
| **Total** | **5226** | **4225** | **3851** | **2645** | **905** |
