# Supplementary material for: Gene discovery in EST sequences from the wheat leaf rust fungus Puccinia triticina sexual spores, asexual spores and haustoria, compared to other rust and corn smut fungi
Source: BMC Genomics. 2011 Mar 24;12:161. doi: 10.1186/1471-2164-12-161 (PMC3074555; doi:10.1186/1471-2164-12-161)
Supplement: Additional file 4 — Similarity searches of Pt unigene sequences to various databases. This table shows the number of unigenes matching the various databases within certain ranges of e-values. [file 1471-2164-12-161-S4.DOC]

**Additional file 4. Similarity searches of *Pt* unigene sequences to various databases**

|  | **nr ^a^** | **EST_other** | **COGEME** | **UniProt** |
| --- | --- | --- | --- | --- |
| **e_value** | **BLASTX** | **TBLASTX** | **TBLASTX** | **BLASTX** |
| < = -100 | 157 (171) | 627 (641) | 54 (54) | 141 (153) |
| -100 <x≤ -50 | 497 (514) | 1248 (1358) | 29 (244) | 468 (485) |
| -50 <x≤ -20 | 987 (1030) | 1078 (1290) | 567 (596) | 939 (976) |
| -20 <x≤ -5 | 986 (1124) | 754 (2565) | 833 (2334) | 980 (1093) |
| TOTAL | 2627 (2839) | 3707 (5854) | 1693 (3228) | 2528 (2707) |

^a^ the 6,308 most-likely *Pt* unigene subset (as well as the complete 13,328 *Pt* unigene sequences: numbers in parentheses) were compared to several major databases using the algorithms indicated. nr, non-redundant sequence database at NCBI (<http://www.ncbi.nlm.nih.gov/nuccore>); EST-other, EST database at NCBI; COGEME, phytopathogenic fungi and oomycete-specific EST database [29]; <http://cogeme.ex.ac.uk/>); UniProt, protein database at NCBI.
