# Supplementary figures and images for: Gene discovery in EST sequences from the wheat leaf rust fungus Puccinia triticina sexual spores, asexual spores and haustoria, compared to other rust and corn smut fungi
Source: BMC Genomics. 2011 Mar 24;12:161. doi: 10.1186/1471-2164-12-161 (PMC3074555; doi:10.1186/1471-2164-12-161)

# PtContig5547 family (ClustalW pile-up without outgroup 'um10816' in support of phylogram Fig. 4A)

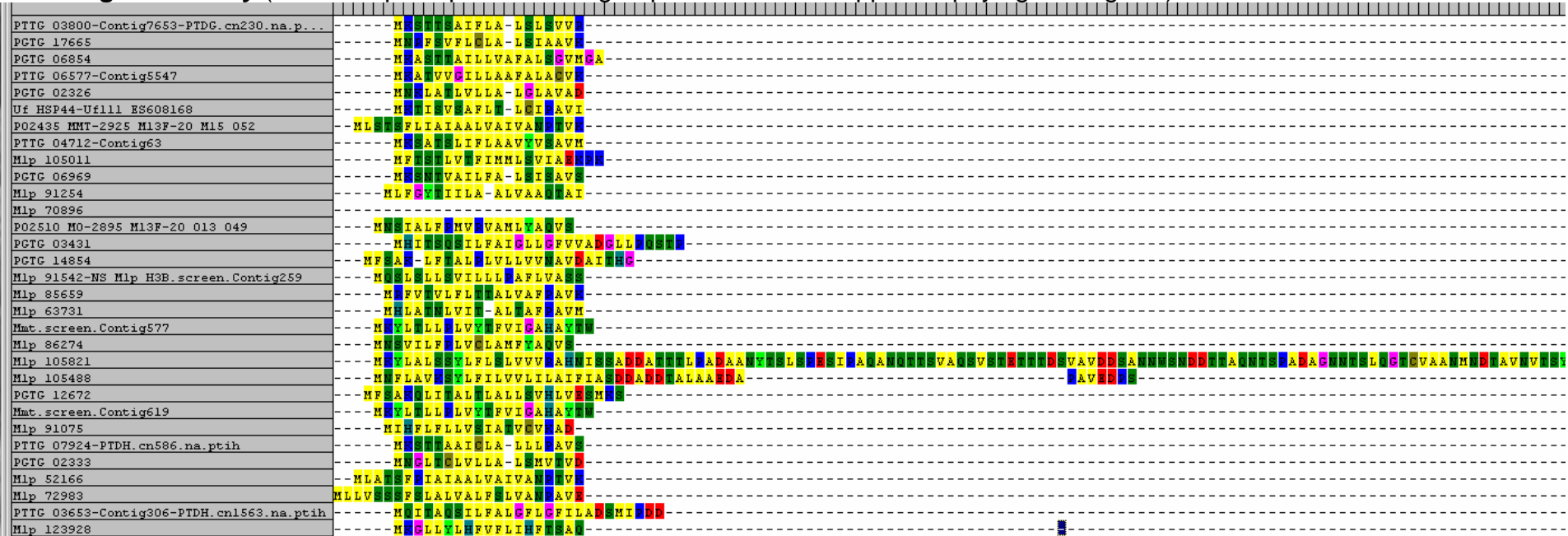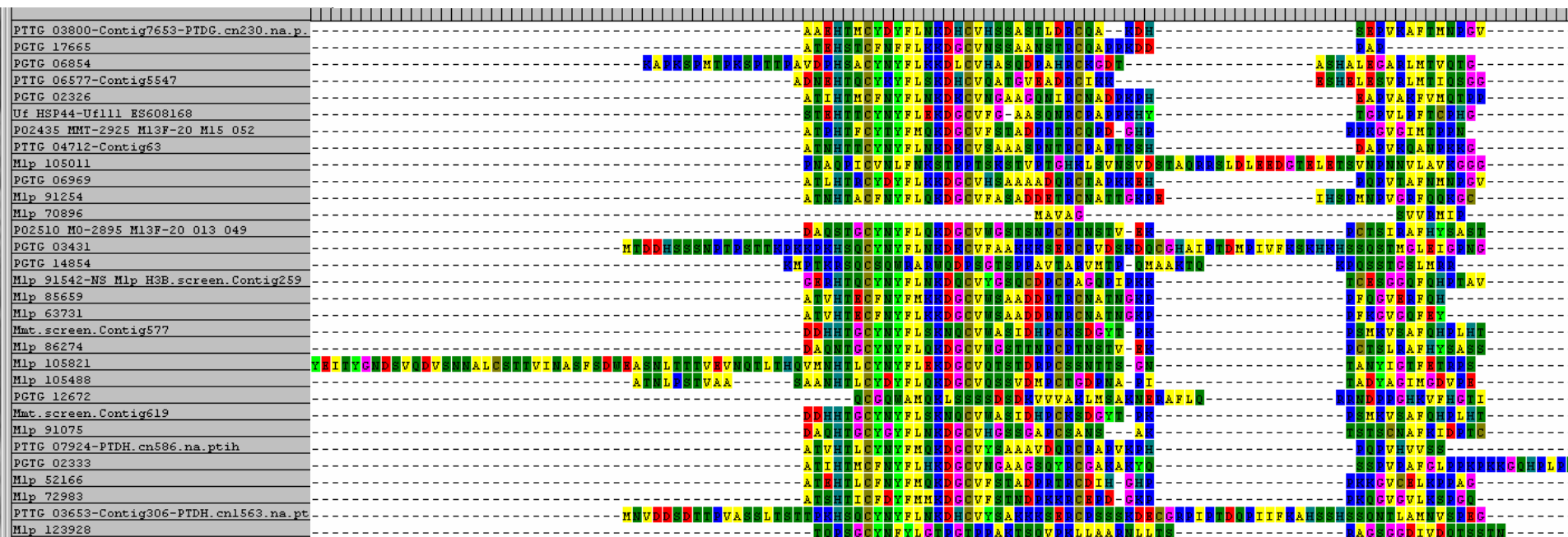

Supplement: Additional file 10 — ClustalW alignment of two families of predicted (small) secreted proteins (SSPs). The alignment was used for the construction of the phylograms in Figure 4. [file 1471-2164-12-161-S10.PDF]
